# On-Chip Compressive Sensing with a Single-Photon Avalanche Diode Array

**DOI:** 10.3390/s23094417

**Published:** 2023-04-30

**Authors:** Chenxi Qiu, Peng Wang, Xiangshun Kong, Feng Yan, Cheng Mao, Tao Yue, Xuemei Hu

**Affiliations:** School of Electrical Science and Engineering, Nanjing University, Nanjing 210023, China; chenxiqiu@smail.nju.edu.cn (C.Q.); wpeng@smail.nju.edu.cn (P.W.); yuetao@nju.edu.cn (T.Y.)

**Keywords:** single-photon avalanche diode, compressed sensing, efficient scene perception

## Abstract

Single-photon avalanche diodes (SPADs) are novel image sensors that record photons at extremely high sensitivity. To reduce both the required sensor area for readout circuits and the data throughput for SPAD array, in this paper, we propose a snapshot compressive sensing single-photon avalanche diode (CS-SPAD) sensor which can realize on-chip snapshot-type spatial compressive imaging in a compact form. Taking advantage of the digital counting nature of SPAD sensing, we propose to design the circuit connection between the sensing unit and the readout electronics for compressive sensing. To process the compressively sensed data, we propose a convolution neural-network-based algorithm dubbed CSSPAD-Net which could realize both high-fidelity scene reconstruction and classification. To demonstrate our method, we design and fabricate a CS-SPAD sensor chip, build a prototype imaging system, and demonstrate the proposed on-chip snapshot compressive sensing method on the MINIST dataset and real handwritten digital images, with both qualitative and quantitative results.

## 1. Introduction

Due to its digitized sensing nature of light and high sensitivity, single-photon avalanche diode (SPAD) imagers have been applied in a variety of applications, such as low light imaging, high dynamic range imaging, etc. [1,2,3]. Realizing a two-dimensional SPAD sensor with a large pixel number is a long-pursued goal, which could be readily applied for computer vision tasks [4,5,6,7,8]. However, due to the working principle of SPAD, the requirement of the self-contained controlling circuit, in-pixel or periphery data acquisition, or storage memory become the bottleneck for fabricating the SPAD arrays with a large pixel number [9]. Furthermore, the growing requirement of bandwidth with the increase of the pixel number poses another challenge. To realize format SPAD array, either column-wise shared periphery circuits [10] or 3D stacking technology [11] is used, facing the trade-off between the readout speed and high cost, with non-reduced data bandwidth. Therefore, how to efficiently realize a large-format SPAD array is still an unresolved problem. Since 2006, the theory of compressed sensing (CS) [12,13,14] has been proposed for efficient data sampling and high-fidelity sparse recovery. Implementing the idea of CS on the CMOS (complementary metal–oxide–semiconductor) sensor chip has been recently proposed [15,16,17,18,19,20,21] and shows promising data throughput reduction on conventional sensing techniques.

In this paper, to realize efficient SPAD array sensing, we introduce compressed imaging technology to the design of the SPAD array. Our CS-SPAD array chip could implement compressive sensing in a snapshot way and reduce the total bandwidth of imaging data to 25%. Besides, the required sensor area for readout electronics with memory is reduced by 25%.

**Figure 1:**
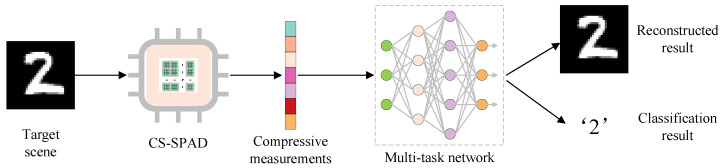
Pipeline of the proposed CS-SPAD sensing method.

The entire pipeline of the proposed scheme is shown in Figure 1. Specifically, we propose to realize compressive sensing on a chip with a 32 × 32 SPAD array. Through introducing different electronic connections between the readout electronics and the sensing pixels, photons collected by different combinations of sensor pixels can be captured in a summation way. The connections are set according to the compressive sensing matrix and the measurement of each readout electronics corresponds to a CS measurement. Furthermore, to retrieve information from the compressively sensed data of the CS-SPAD imaging sensor, we propose a CSSPAD-Net which could realize both image recovery and image classification based on the captured data of the CS-SPAD array. To demonstrate the proposed method, we design and fabricate the CS-SPAD sensor array, build a prototype imaging system based on the chip, and realize both image recovery and classification based on the output of the sensor, with high fidelity. In all, with the proposed CS-SPAD sensor, we could realize SPAD array sensing efficiently, i.e., with 25% data throughput and 25% periphery electronics required for two-dimensional sensing. The performance of imaging and classification of the CS-SPAD imaging system is demonstrated on the MNIST [22] dataset and real handwritten digital images, both quantitatively and qualitatively.

## 2. Related Work

Large-format SPAD imaging arrays have been a long-pursued goal [9,10,11,23,24,25], with wide application in low-light imaging [1,26], high-dynamic-range imaging [2,3], 3D imaging [1,27], etc. To realize the photon-resolving working principle of SPAD, the peripheral circuit, including the quenching circuit, readout electronics, etc., for generating the photon counting results are required and occupy a large area of the SPAD sensor, preventing the implementation of a large-format SPAD imager. Commonly, to realize large-format SPAD arrays, the peripheral circuits or counting electronics with memory are commonly shared among columns or rows of pixels [10], facing a trade-off between the readout speed and required sensor area. To realize large-format SPAD imaging without sharing the readout electronics, 3D stacking technology is introduced and large-format SPAD imaging can be realized, at the expense of high cost [11,28]. Furthermore, the requirement of data bandwidth are not reduced, becoming a potential bottleneck for large-format SPAD arrays. Thus, how to realize a SPAD imager with an efficient sensor area is still an open problem.

Compressive sensing [12], firstly proposed in 2006, provides an elegant solution to reduce the required data transmission bandwidth through sampling the scene information with a sub-Nyquist sampling rate and compressive reconstruction, taking full advantage of nature image redundancy. Mathematically, the scene redundancy is modeled with sparsity in the image transform domain, e.g., Fourier or wavelet transform domain [29], learned transform domain [30,31], etc. Through restricting the sparseness of the signal in the transform domain, the original signal can be recovered with high fidelity from the compressive measurement. Based on the compressive sensing theory, efficient imaging technology based on an optical system is proposed, such as single-pixel imaging [32], ghost imaging [33], low-light imaging [34] and mid-infrared imaging [35], which could largely reduce the required data transmission bandwidth.

Sun et al. [36] proposed to build a discrete micromirror device modulation-based compressive SPAD imaging system to realize a high-resolution SPAD imager. However, the requirement of a spatial light modulator leads to a large increase of the imaging complexity. Besides, multiple measurements are required for different compressed codes, which largely restricts the speed of compression acquisition and prevents real-time imaging.

In order to avoid complex optical systems, some on-chip compressed sensing schemes have been proposed in conventional imaging sensors, which could realize efficient data reading and reduced data bandwidth. Conventional CMOS image sensor converts light intensity into electrical signals for each pixel individually, while CS CMOS image sensors only sample a small set of random pixel summations [15,16,17,18,19,20,21], which can reduce the size of output data, analog to digital conversion (ADC) operations and the sensor power consumption.

In this paper, we propose to implement compressive sensing on the SPAD sensor array, which could realize compressive sensing on the chip and help to reduce the data throughput and required sensor area for data reading and memory. Specifically, we design the electrical circuits between the sensing unit and the readout electronics to realize the compressive sensing process. Each set of readout electronics is designed to count the pixels in a local unit and integrate the process of data compression into the chip through the local coupling of pixels on the chip, which reduces the sensor area required for data storage and data throughput to 25%.

To demonstrate our methods, we propose CSSPAD-Net to process the captured data by the CS SPAD chip, fabricate and tape out the CS-SPAD image sensor, and build a prototype imaging system. The effectiveness and efficiency of the proposed CS-SPAD sensor are demonstrated, both quantitatively and qualitatively, on the MNIST dataset [22] or real handwritten digital images. We will introduce the details of our method in the methods section.

## 3. Methods

In order to realize efficient SPAD array sensing, we design a novel compressive sensing SPAD array which can directly record the compressively sensed data. In the decoding process, we propose a neural network designed to directly process the compressively sensed data, which can reconstruct the scene and realize classification. This section will introduce our methods, including the proposed compressive imaging chip design and information processing network architecture for compressed data.

### 3.1. Snapshot Compressed Imaging Chip

#### 3.1.1. Basic Compressed Coding Unit

**Figure 2:**
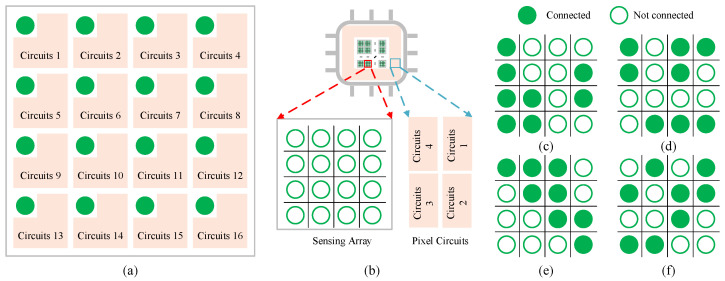
(**a**) Classic sensor array (**b**), basic compressed sensing imaging unit of CS-SPAD, (**c**–**f**) four different CS connection settings.

The basic compressed coding unit directly records the readout of the sensor after exposure by linking readout electronics (with memory) to each pixel, as shown in Figure 2a.

**Figure 3:**
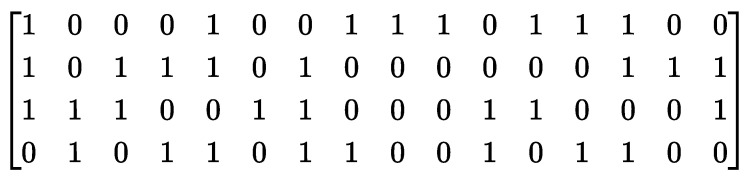
An example of a CS-SPAD measurement matrix.

In this paper, we propose to implement compressive sensing in a block-wise way, i.e., we design n×n(n=4) pixel block as the basic compressed coding unit and only use m(m=1∼4) readout electronic (with memory) to record the 0−1 weighted sums of intensity distributions from n×n pixels. In other words, the value of each readout electronics (with memory) is the sum of the random pixels in the n×n area. This operation can be abstracted into the basic formula of compressed sensing:

(1) $$y=Ax+w;\;A\in R^{m\times n^{2}},\;x\in R^{n^{2}\times 1},\;y\in R^{m},$$

where $A\in R^{m\times n^{2}}$ is the compressive measurement matrix consisting of 0 and 1, which denotes the designed circuit connection state between the readout circuits and the pixels in each n×n pixel unit. The connection settings within each block of pixels are shown in Figure 2c–f; if the readout circuit is connected with the pixel, the corresponding value in the measurement matrix is 1 and the readout circuit will collect the output of this pixel. If not, it is 0 and the readout circuit does not record the readout of this pixel. For example, in the basic compressed coding unit of our CS-SPAD, the measurement matrix *A* is shown in Figure 3.

$x\in R^{n^{2}\times 1}$ is the compressed sensing signal to be sampled and is also equivalent to the number of photons arriving at the pixel within an exposure time in a basic compressed coding unit. $y\in R^{m}$ is the sampled signal in compressed sensing in a basic compressed coding unit.

#### 3.1.2. CS-SPAD

Figure 2b shows the overall layout of the CS-SPAD chip we designed. The entire pixel array of CS-SPAD consists of the basic compressed coding unit described above. For each pixel in the basic compressed coding unit, we use a single-photon avalanche diode as a solid-state photodetector to record the number of photons arriving at each pixel. The readout is the sum of the photon counts of all its connected pixels. For each 4 × 4 SPAD local block, four readout circuits are required to capture the compressively sampled data, i.e., the use of readout circuits is also reduced to 25%.

### 3.2. Information Processing Architecture Based on Convolution Neural Network

**Figure 4:**
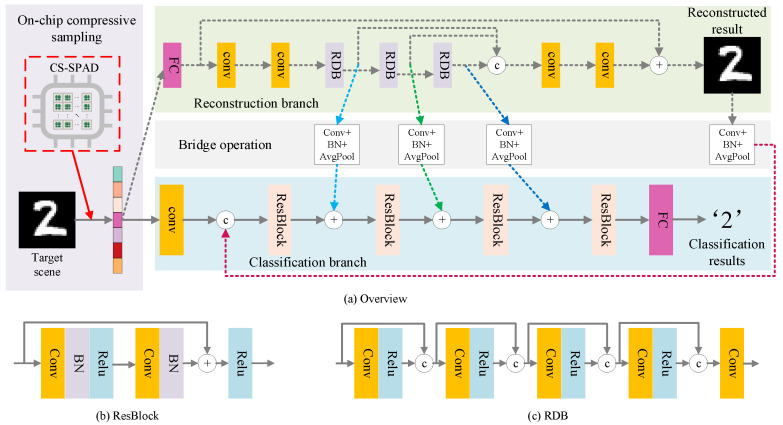
(**a**) Overview of CSSPAD-Net, (**b**) the structure of residual block (Resblock), (**c**) the structure of residual dense block (RDB).

We propose CSSPAD-Net to realize multi-task processing upon the compressed measurements from our chip, realizing both image reconstruction and classification, as shown in Figure 4.

#### 3.2.1. Reconstruction Branch

The proposed CS-SPAD sensor compressed the data by dividing the image, of which the pixel resolution is 32×32, into a basic processing unit. In the basic processing unit, 4×4 pixels are compressed into 1×4 values. Based on the basic processing unit, a fully connected layer, for which the number of input channels is 4 and the number of output channels is 16, is used for the recovery of compressed data dimensions in a basic processing unit. All basic CS processing units in the same compressed data are upsampled by using this fully connected layer. Once the above steps have been completed, the initial reconstruction of the scene is completed through tilling the reconstructed block images from different CS pixel units. We further refine the initially reconstructed image by using convolutional layers to eliminate the block artifacts caused by the block processing of the CS-SPAD. For further utilization of the statistical prior of nature images, we utilize a similar structure through a global–local residual learning way. Global residual learning [37] could enforce the overall goal of the network to learn the residual details of the initially reconstructed image and largely improve the learning difficulty compared to directly learning the image itself [38], and we introduce the local residual learning in the residual dense block (RDB) to further helps the fusion of deep and shallow features in the network.

#### 3.2.2. Classification Branch

To realize efficient perception with the proposed CS-SPAD sensor, we propose to realize classification besides reconstruction. In the classification branch, we propose to use four residual blocks as the main body of the classification branch and a linear layer for final classification for efficiency. As is well known, in the image reconstruction network, the shallow layers of the network contain more low-level details and the deep layer of the network contains more high-level features [39]. Bridge operations between the two branches are introduced to guarantee the information fusion among different tasks.

#### 3.2.3. Implementation Details

We synchronously train the reconstruction branch and the classification branch in our CSSPAD-Net together. The loss function in the reconstruction branch is the mean square error (MSE) loss $L^{\mathrm{MSE}}$, and we set the learning rate to 0.0004. Given reconstructed image $x^{\mathrm{rec}}$ and the ground truth image *x* with *N* pixels, the MSE loss can be calculated as:

(2) $$\begin{array}{c} L^{\mathrm{MSE}}=\frac{1}{N}{∥x^{\mathrm{rec}}-x∥}_{F}^{2}. \end{array}$$

where ||F(.)|| is the Frobenius norm. In the classification branch, the loss function is the cross entropy (CE) loss and the learning rate is set to 0.1. Given predicted vector $x^{\mathrm{pred}}=\left[x_{1},x_{2},\ldots ,x_{C}\right]$ with *C* classes and the ground truth class *j*, the CE loss can be calculated as:

(3) $$\begin{array}{c} L^{\mathrm{CE}}=-\log\frac{e^{x_{j}^{\mathrm{pred}}}}{\sum_{i=1}^{C}e^{x_{i}^{\mathrm{pred}}}}. \end{array}$$

We train our CSSPAD-Net on Nvidia GeForce RTX 2080 for 200 epochs and the learning rate of both is reduced by half every 50 epochs. The batch size is set to 128. The Adam optimizer [40] is adopted with $\beta_{1}=0.9$, $\beta_{2}=0.999$ and $\epsilon =1\times 10^{-8}$.

## 4. Experiments

We verify the CS-SPAD sensor in real scenes and use CSSPAD-Net to complete scene reconstruction and perceptual classification. Unlike commercial cameras that integrate focusing and control systems, the CS-SPAD we proposed is just a computational sensor with a photoelectric conversion function, which requires an additional focusing lens and control system to cooperate with the CS-SPAD sensor. In this section, we first introduce our CS-SPAD sensor chip and the optical system, after which we will demonstrate the effectiveness of the CS-SPAD sensor and the proposed CSSPAD-Net with experimental results on MNIST data [22].

### 4.1. Prototype CS-SPAD Sensor Chip and the Optical System

**Figure 5:**
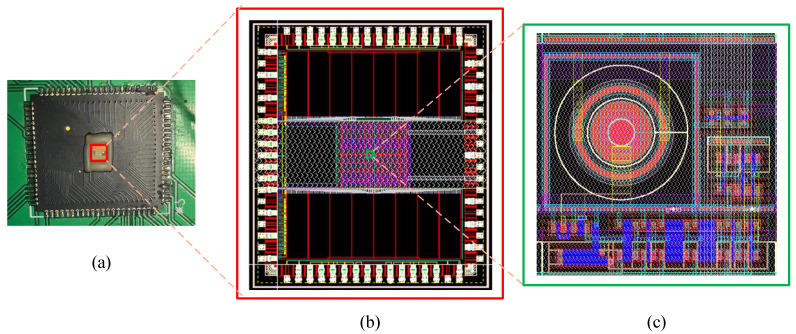
(**a**) CS SPAD chip overview, (**b**) the diagram of CS-SPAD sensor chip design, (**c**) single pixel layout.

We designed a 32 × 32 CS-SPAD sensor chip to realize on-chip snapshot-type spatial compressive imaging, as shown in Figure 5a. The system mainly includes three parts: a 32 × 32 SPAD detector array, readout circuits, and address decoding circuits. The 32 × 32 SPAD detector circuit includes the SPAD detector, the corresponding gating quenching circuit, and the logic circuit required for compressive sensing. The readout circuit includes a pulse shaper, a transmission circuit, a 12-bit counter circuit, and a 12-bit latch circuit. The address decoding circuit uses two identical 8 × 16 two-stage decoding modules, which work synchronously and transmit data through two 12-bit IO data ports.

**Figure 6:**
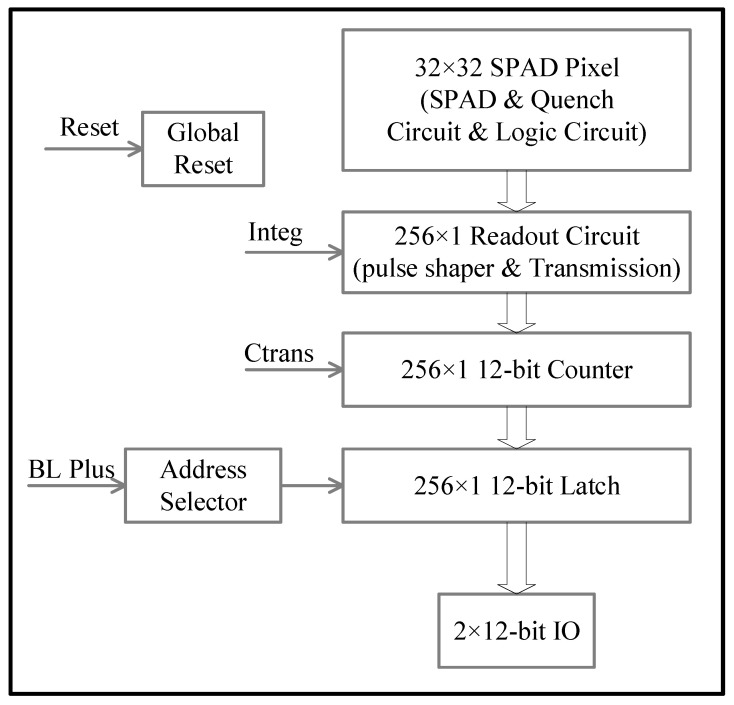
The CS SPAD chip architecture.

The chip system architecture is shown in Figure 6, consisting of three main parts: a 32 × 32 SPAD detector array, 256-row readout circuits, and an address decoding circuit. The 32 × 32 SPAD detector circuit includes the SPAD detector, the corresponding gating quenching circuit, and the logic circuits for multiple “wired AND” operations required by compressed sensing. The row readout circuit includes a shaping circuit, a transmission circuit for integrating time, a 12-bit counter circuit, and a 12 bit-buffer circuit. The address decoding circuit adopts two identical 8 × 16 two-stage decoding modules, which work synchronously and transmit data through two 12-bit IO data ports.

The chip’s internal structure and the pixel layout are shown in Figure 5b,c, respectively. We adopt the 0.18 μm 1P6M CMOS technology, and the SPAD pixel size is 15 μm. The array size is 32 × 32 and the bit depth of the counter is 12 bit. The calibrated dark count of the proposed CS-SPAD is 200 cps at room temperature, i.e., 300 K. The dead time of the sensor is about 20 ns. Our CS-SPAD works in avalanche mode with single-photon sensitivity and the quantum efficiency is about 15%. The performance summary of the CS-SPAD sensor chip is shown in Table 1.

Additionally, as shown in Table 2, we compared different SPAD-based imagers or imaging systems with our CS-SPAD. We propose the on-chip compressed sensing method that performs compressed sampling directly on the chip, which can effectively avoid the complexity of the optical system.

**Figure 7:**
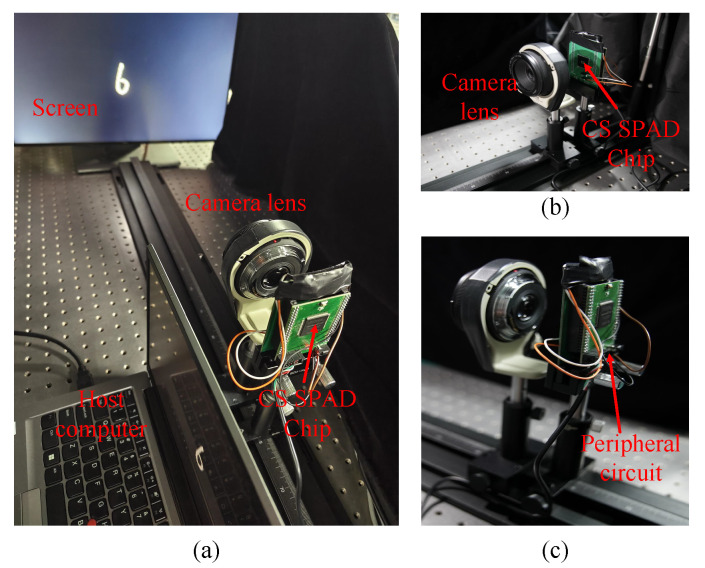
(**a**) Overview of the prototype imaging system, (**b**) the detail of camera lens and CS SPAD chip, (**c**) the detail of peripheral circuits.

As shown in Figure 7, we build a prototype imaging system based on the proposed CS-SPAD sensor. From left to right are the target scene, the lens used for focusing, the CS-SPAD sensor, and the host computer for controlling the automatic execution of the system. The working pipeline of the CS-SPAD imaging is as follows: first, the host computer controls the display to display a pattern to be sampled on the monitor, then the CSSPAD is exposed, and finally the compressed data sampled by the CS-SPAD sensor are read out and the reconstruction and classification results can be implemented with the trained network on the host computer.

### 4.2. Experiment Results

#### 4.2.1. Dataset and End-to-End Network Training

We evaluate the CS-SPAD sensor and the CSSPAD-Net on the MNIST [22] dataset, a dataset of handwritten digits for classification tasks. We use the full MNIST dataset with 60,000 training images and 10,000 test images for the validation of our entire system.

#### 4.2.2. Simulation and CSSPAD Sampling

**Figure 8:**
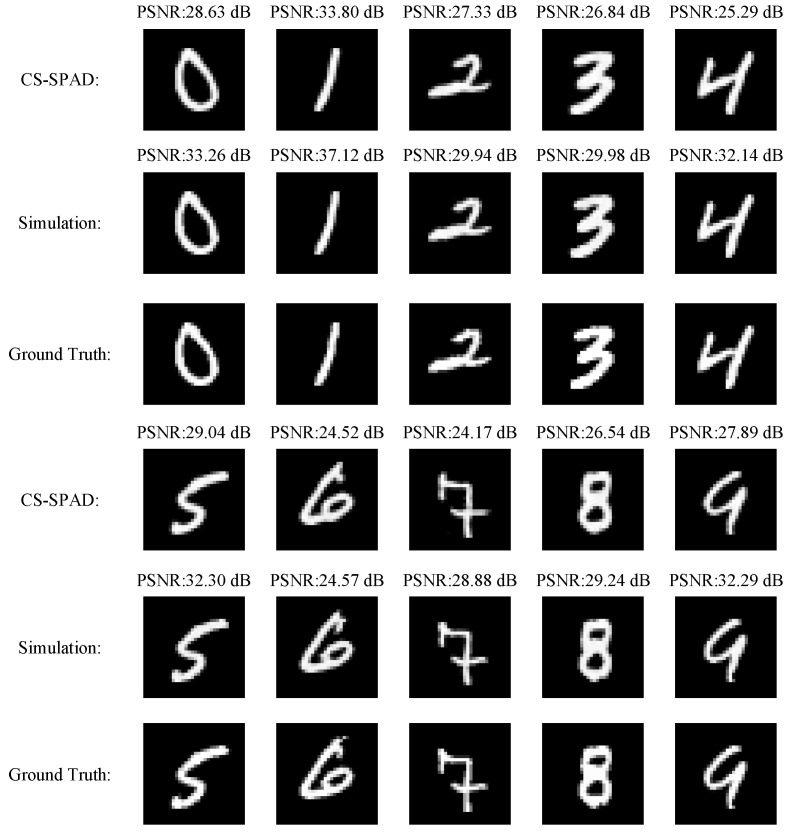
Reconstructed results by CSSPAD-Net.

For quantitative evaluation, with the test dataset, we display the images on the screen, capture the compressed data with the CS-SPAD sensor, reconstruct the image with the CSSPAD-Net, and calculate the reconstruction metrics with the reconstructed image and the corresponding projected image. The reconstruction and the classification results are shown in Table 3, we use PSNR (peak signal-to-noise ratio) and SSIM (structural similarity index measure) [51] to evaluate the quality of the reconstruction. As shown, our method could realize high classification accuracy and preserve almost all the details with high SSIM metrics. For quantitative evaluation, we further show the reconstruction result of the reconstruction branch in CSSPAD-Net in Figure 8. In the figure, “CS-SPAD” indicates that the acquisition is completed by the CS-SPAD chip, and the reconstruction process is completed on the GPU. Moreover, “simulation” indicates that the acquisition process simulates the CS-SPAD acquisition process, and the reconstruction is also completed on the GPU. As is shown, the structural details of different digits are elegantly recovered with fine details.

**Figure 9:**
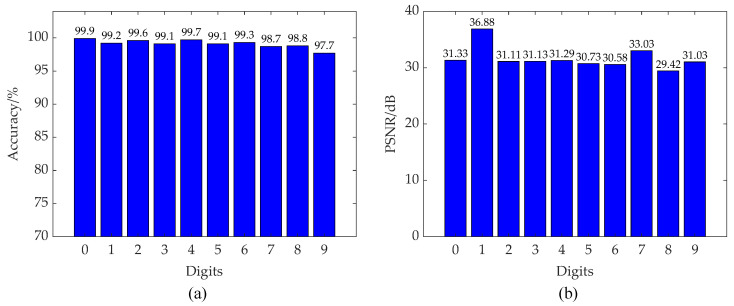
Reconstruction and classification results for different digital number categories by CSSPAD-Net. (**a**) Reconstruction PSNR with different digital number categories and (**b**) classification accuracy with different digital number categories.

We also analyzed the reconstruction quality and classification accuracy of different categories of digital numbers, as shown in Figure 9 Although the PSNR of different categories of the reconstructed digital numbers fluctuates a little, it has little effect on the classification results of the classification branch.

#### 4.2.3. Real Handwritten Data Experiment

**Figure 10:**
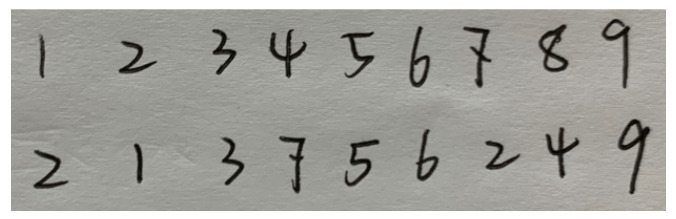
Example of real handwritten digits.

**Figure 11:**
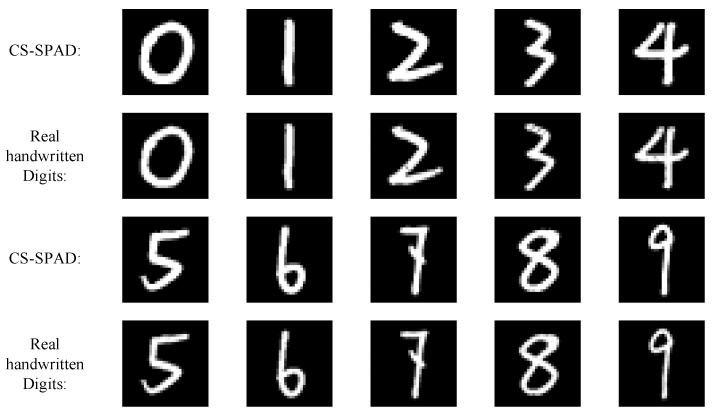
Real handwritten digits: reconstructed results.

Furthermore, as shown in Figure 10, we handwrite more digits, not from MNIST, for verification of our entire system. We use the same model trained on the MNIST dataset to classify and reconstruct the real handwritten digits. The visual display of the reconstructed results are shown in Figure 11, as shown, our methods could realize elegant reconstruction with the compressed results captured by the CS-SPAD sensor.

## 5. Conclusions

In this paper, we propose CS-SPAD to realize on-chip spatial compressive imaging, which could reduce the required sensor area for readout electronics and the data throughput to 25%. CSSPAD-Net is further proposed to recover images from compressively sampled data and implement high-accuracy perceptual classification. We taped out the CS-SPAD array and built a prototype imaging system to demonstrate the effectiveness of the sensor. Quantitative and qualitative experiments on MNIST dataset [22] and real hand-written digits were conducted to demonstrate the effectiveness and efficiency of our proposed CS-SPAD imaging sensor. For future work, we plan to further improve the performance of the CS-SPAD imaging chip and extend the on-chip CS idea to 3D imaging. Specifically, since existing research on compressed sensing [52,53,54,55,56] have shown that the joint optimization of the reconstruction network and the compressive sensing matrix can greatly improve the imaging efficiency, we plan to introduce the end-to-end optimization of the sensing matrix and reconstruction algorithm to further improve the CS imaging efficiency of our CS-SPAD imager. Beyond that, since a large-format SPAD array with a time-to-digital converter (TDC) module is in high demand for 3D imaging, which encounters more severe challenges of high data bandwidth and large sensor area for peripheral circuits of TDC, we plan to further develop an on-chip CS-SPAD with a TDC module, based on the proposed CS-SPAD method, to realize efficient 3D CS detection.

## Figures and Tables

**Table 1:** Performance summary of the 32 × 32 CS-SPAD sensor chip.

Parameter	Value
Technology	0.18 μm 1P6M CMOS
Chip size	2.9 mm × 3.3 mm
Array size	32 × 32
Pixel size	15 μm
Counter width	12 bit
Dark counts rate	200 cps
Dead time	20 ns
Power supply	1.8 V (Digital)/3.3 V (Analog)
	/12 V (SPAD cathode voltage)
Power consumption	10 mW@12 V SPAD cathode voltage

**Table 2:** Overview of recently developed SPAD imagers or imaging systems.

SPAD Sensor or Imaging System	Year	CS	CS Methods	SPAD Array	Technology (nm)	Pixel Size (μm)	Dark Count Rate (cps)
[41]	2015	No	None	256×256	130	4.2	30.8
[42]	2016	No	None	160×160	350	15	580
[43]	2016	No	None	72×60	180	15	2.3
[1]	2016	Yes	Optical	32 × 32	350	150	100
[44]	2017	No	None	256×256	130	14.1	6200
[45]	2017	No	None	256×1	350	17.1	1286.6
[46]	2018	No	None	32×32	180	17	113
[36]	2018	Yes	Optical	64×32	/	30	150
[47]	2018	No	None	512×512	180	6	7.5
[48]	2019	No	None	256×256	40/90	9.2/38.4	20
[10]	2020	No	None	1024×1000	180	9.4	0.4/2.0
[49]	2020	No	None	1200×900	65	6	100
[11]	2021	No	None	2072×1548	40/90	6.39	1.8
[50]	2021	No	None	189×600	40/90	10	2000
[27]	2022	Yes	Simulation	/	/	/	/
[23]	2022	No	None	500×500	180	16.38	10.2
[35]	2023	Yes	Optical	1×1	/	180	100
[24]	2023	No	None	512×1	180	26.2	<100
Ours	2023	Yes	On-chip	32 × 32	180	15	200

**Table 3:** Accuracy, PSNR, and SSIM of two datasets.

Dataset	Average PSNR/dB	Average SSIM	Accuracy/%
CSSPAD	27.3039	0.9819	99.22
Simulation	31.6760	0.9930	99.31

## Data Availability

The Mnist [22] data presented in this study are openly available and can be founed at http://yann.lecun.com/exdb/mnist/, accessed on 27 April 2023.
